# Aquaporin 3 promotes epithelial-mesenchymal transition in gastric cancer

**DOI:** 10.1186/1756-9966-33-38

**Published:** 2014-05-03

**Authors:** Jia Chen, Tao Wang, Yang-Chun Zhou, Fei Gao, Zhi-Hong Zhang, Hao Xu, Shou-Lin Wang, Li-Zong Shen

**Affiliations:** 1 Division of Gastrointestinal Surgery, Department of General Surgery, First Affiliated Hospital, Nanjing Medical University, 210029 Nanjing, Jiangsu, China; 2 Department of Internal Medicine, First Affiliated Hospital, Nanjing Medical University, Nanjing, Jiangsu, China; 3 Department of Pathology, First Affiliated Hospital, Nanjing Medical University, Nanjing, Jiangsu, China; 4 School of Public Health, Nanjing Medical University, Nanjing, Jiangsu, China

**Keywords:** Gastric cancer, Aquaporin 3, Epithelial-mesenchymal transition, E-cadherin, Vimentin

## Abstract

**Background:**

Gastric carcinoma (GC) is a common and lethal malignancy, and epithelial-mesenchymal transition (EMT) is believed to contribute to invasive and metastatic tumor growth. Aquaporin 3 (AQP3) is overexpressed in human GC tissues, while human epidermal growth factor (EGF) and hepatocyte growth factor, which can induce EMT, are able to up-regulate AQP3 expression, subsequently promoting GC cell migration and proliferation. The purpose of this study was to investigate the effects of AQP3 on EMT in human GC.

**Methods:**

AQP3 and EMT-related proteins were detected by immunohistochemistry in human GC specimens and their clinical significance evaluated. AQP3 knockdown was attempted using small interfering RNAs, while EGF was used to up-regulate AQP3 expression. Western blotting, real-time quantitative polymerase chain reaction assays and immunofluorescence were used to evaluate changes in expression of AQP3 and EMT-related proteins in the SGC7901 and MGC803 human GC cell lines.

**Results:**

AQP3 up-expression was associated with EMT-related proteins in human GC specimens, which correlated with poor prognosis for GC. AQP3 modulated GC cell proliferation, migration and invasion in vitro, and induced E-cadherin repression. AQP3 also up-regulated the expression of vimentin and fibronectin in vitro. The PI3K/AKT/SNAIL signaling pathway was likely involved in the induction of EMT by AQP3 in GC.

**Conclusions:**

AQP3 promotes EMT in human cases of GC, allowing us to understand the mechanisms of AQP3 in GC progression, thus providing a potential strategy for its treatment.

## Background

Gastric carcinoma (GC) remains one of the most common and lethal malignancies worldwide [1]. Despite radical surgery and chemotherapy, invasion and metastasis result in very low survival rates [2]. The mechanisms underlying GC invasion and metastasis remain to be elucidated. GC invasion or metastasis is a multistep process that encompasses cancer cell invasion into surrounding tissues, entry into the systemic circulation, survival in the circulatory system, adhesion to endothelial cells, extravasation at distant organs, and the formation of secondary tumors [2,3].

There is a growing understanding that epithelial-mesenchymal transition (EMT) contributes to invasion and metastasis [4-6]. The term EMT refers to a complex molecular and cellular process by which epithelial cells shed certain characteristics (such as cell-cell adhesion, planar and apical-basal polarity, and lack of motility), and acquire mesenchymal features (motility, invasiveness, and resistance to apoptosis) [7]. EMT plays key roles in embryonic development and is recognized as an important contributor to the pathogenesis of cancer and other human diseases [8,9]. During EMT, expression levels of the adhesion molecule E-cadherin are decreased, whereas N-cadherin and vimentin levels are increased. These molecular alterations possibly cause dysfunctional cell-cell adhesion and loss of cell-cell junctions, thereby allowing dissemination of tumor cells from the primary sites. It is widely accepted that EMT contributes to invasion, metastatic dissemination, and acquired resistance to therapy [10,11].

Aquaporins (AQPs) are a family of small, integral membrane proteins that transport water and, in some cases, water and glycerol. Apart from these physiological functions [12], accumulating evidence further implicates the role of AQPs in cell migration and proliferation [13-15]. Previously, we showed that GC tissues expressed higher levels of aquaporin 3 (AQP3) compared with that in normal mucosa. Additionally, AQP3 expression was associated with histological classification, lymph node metastasis, and lymphovascular invasion [16], indicating the involvement of AQP3 in the carcinogenesis and progression of GC. Human epidermal growth factor (EGF) [17] and hepatocyte growth factor (HGF) [18] up-regulate AQP3 expression via the extracellular signal-regulated kinase (ERK) pathway, then promote cell migration and proliferation in vitro, suggesting that AQP3 could be a potentially important determinant of tumor growth and the spread of GC.

Little is known about the mechanisms of AQP3 with respect to GC invasion and metastasis. It is well understood that EMT can be induced by a large variety of stimuli during tumor progression [10]. Studies have shown that HGF and EGF can induce EMT in hepatocellular carcinoma and colon cancer respectively [19,20]. Recently, we showed that AQP3 positively regulates matrix metalloproteinases (MMPs) in GC cells [21], however up-regulation of MMPs is a characteristic of EMT [22]. We speculated that AQP3 might induce EMT and consequently promote GC cell migration and metastasis. We investigated expression levels of AQP3 and EMT-related proteins in human GC tissues, evaluating their clinical significance and cross-correlation. We also studied the effects of AQP3 on EMT-related proteins and the involved signaling pathway in human GC cells.

## Materials and methods

### Human gastric tissue specimens

Patients diagnosed with gastric adenocarcinoma (n = 89; median age, 56 years; range, 35–75 years) between June 2007 and September 2008 at the Department of General Surgery, First Affiliated Hospital, Nanjing Medical University, were randomly enrolled in this study. All patients were diagnosed pathologically according to the American Joint Committee on Cancer (AJCC) criteria. None of these patients had received chemotherapy or radiotherapy before surgery. Samples of tumor and corresponding non-cancerous tissue from all patients were collected immediately after resection and snap frozen in liquid nitrogen. These human gastric tissue specimens had been used in our previous study [16]. All patients were followed up until September 2013, with a median follow-up of 60 months. Overall survival (OS) was defined as the interval between the dates of surgery and death. The correlation between expression of AQP3, E-cadherin or vimentin, and clinicopathological characteristics of patients was evaluated. These characteristics are listed in Table 1. No cases with distant metastasis were observed in this study. This study was approved by the Nanjing Medical University Institutional Review Board. Written consent was given by the patients for their information and samples to be stored in the hospital database and used for research. This study was also in compliance with the Helsinki Declaration.

**Table 1:** Correlation between AQP3, E-cadherin,vimentin expression and clinicopathological features in GC

**Clinicopathological features**	**n**	**AQP3**			**E-cadherin**			**Vimentin**		
		**+**	**-**	**P-value**	**+**	**-**	**P-value**	**+**	**-**	**P-value**
Age(yr)				0.628			0.825			0.763
≤50	32	22	10		12	20		4	28	
>50	57	43	14		23	34		10	47	
Gender				0.318			0.653			0.363
Male	58	40	18		24	34		11	47	
Female	31	25	6		11	20		3	28	
Lauren classification				0.008			0.659			0.015
Intestinal	54	34	20		20	34		4	50	
Diffuse	35	31	4		15	20		10	25	
Tumor size				0.303			0.816			0.758
<3.0 cm	28	18	10		10	18		5	23	
≥3.0 cm	61	47	14		25	36		9	52	
Tumor location				0.515			0.920			0.880
Upper third	15	10	5		6	9		3	12	
Middle third	26	19	7		11	15		4	22	
Lower third	48	37	9		18	30		5	41	
Depth of tumor invasion				0.511			0.031			0.139
Localized in subserosa	38	13	25		20	18		3	35	
Beyond subserosa	51	22	29		15	36		11	40	
Lymph node metastasis							0.010			0.201
N0	12	4	8	0.002	9	3		0	12	
N1–N3	77	61	16		26	51		14	63	
Lymphovascular invasion				0.044			0.000			0.004
Absence	58	38	20		32	26		4	54	
Presence	31	27	4		4	28		10	21	

### Immunohistochemical detection of AQP3, E-cadherin, and Vimentin

Expression of AQP3, E-cadherin, and vimentin in specimens was determined by immunohistochemistry (IHC) as described previously [18]. A polyclonal rabbit anti-AQP3 antibody was obtained from Santa Cruz Biotechnology (Santa Cruz, CA), and monoclonal antibodies against E-cadherin and vimentin were purchased from Cell Signaling Technology (Beverly, MA). A pathologist scored protein expression as the percentage of positive tumor cells (scale 0–100%) with a staining intensity from 0–3+. Positive IHC expression was defined as >25% staining with an intensity of 2–3 +.

### Cell culture and RNA interference (RNAi)

Human GC cell lines SGC7901 and MGC803 (CBTCCCAS, Shanghai, China) were cultured in RPMI-1640 (Life Technologies, Gibco BRL, Grand Island, NY, USA) supplemented with 10% fetal bovine serum (FBS; Invitrogen), penicillin/streptomycin (1:100 dilution; Sigma, St. Louis, MO), and 4 mM glutamine (Life Technologies, Gibco BRL) at 37°C/5% CO_2_. RNAi assays were conducted according to previous methods [18].

### Western blotting assays

Western blotting was used to detect expression levels of proteins as described previously [18,23]. We used antibodies against AQP3 (Santa Cruz Biotechnology, Santa Cruz, CA), vimentin, E-cadherin, Snail, AKT, phospho-AKT(Ser473) (Cell Signaling Technology, Beverly, MA), fibronectin (R&D systems, Minneapolis, MN), and glyceraldehyde-3-phosphate dehydrogenase (GAPDH) (Beyotime Institute of Biotechnology, Henan, China). Densitometric analysis of proteins was conducted and normalized against GAPDH. The PI3 kinase inhibitor LY294002, was obtained from Cell Signaling Technology (Beverly, MA).

### Real-time quantitative polymerase chain reaction (qPCR) assays

We conducted qPCR assays using previously described protocols [18,23] and the manufacturer’s instructions. We used GAPDH as the reference gene for analysis, with observed expression levels normalized to the expression level of GAPDH. Specific primer sequences were used to amplify targets for AQP3 (5′-CTC GTG AGC CCT GGA TCA AGC-3′ and 5′-AAA GCT GGT TGT CGG CGA AGT-3′), vimentin (5′-ATC TGG ATT CAC TCC CTC TGG TTG-3′ and 5′-CAA GGT CAT CGT GAT GCT GAG AAG-3′), fibronectin (5′-TGT TAT GGA GGA AGC CGA GGT T-3′ and 5′-AGA TCA TGG AGT CTT TAG GAC GCT C-3′), E-cadherin (5′-AAT CCA AAG CCT CAG GTC ATA AAC A-3′ and 5′-GGT TGG GTC GTT GTA CTG AAT GGT), and GAPDH (5′-CGC TGA GTA CGT CGT GGA GTC-3′ and 5′-GCT GAT GAT CTT GAG GCT GTT GTC-3′). All qPCR assays were performed in triplicate.

### Cell proliferation assays

Cells (3 × 10^4^) were seeded in triplicate in 96-well plates and allowed to incubate for 48 h at 37°C/5% CO_2_. An EdU incorporation assay was used to determine cell proliferation according to the manufacturer’s protocol (RiboBio, Guangzhou, China). We used a fluorescence microscope (Olympus Corporation, Tokyo, Japan) to visualize our results. All experiments were performed in triplicate and repeated three times.

### Transwell migration and invasion assays

According to a previous protocol [5], cells (3 × 10^5^ cells/well) were seeded in the upper chambers of 24-well transwell inserts (8.0-μm pore size; Corning, Corning, NY, USA) with 200 μL of RMPI-1640 medium (Beyotime Institute of Biotechnology, Henan, China) containing 1% FBS and 0.2% bovine serum albumin (BSA).

### Immunofluorescence assays

Immunofluorescent staining was performed as previously described [6]. We used the primary antibodies mentioned above, and secondary antibodies were obtained from Beyotime (Beyotime Institute of Biotechnology, Henan, China). Fluorescent images were acquired with a fluorescence microscope (Olympus Corporation, Tokyo, Japan).

### Statistical analysis

Data were expressed as mean ± standard error (SE). In the experiments involving protein expression, values are representative of three independent experiments. We used the χ^2^ and Fisher’s exact test to examine the association between protein expression levels and various clinicopathological parameters. Univariate analysis was performed using the Kaplan–Meier method, and statistical significance between survival curves was assessed by the log rank test. Bivariate correlations between study variables were calculated using Spearman’s rank correlation coefficients. Statistical analyses were completed with SPSS 11.0 (SPSS Inc., Chicago, IL, USA) and a *P*-value less than 0.05 was considered statistically significant.

## Results

### Upregulation of AQP3 and associated EMT-related proteins predict poor prognosis for GC

**Figure 1:**
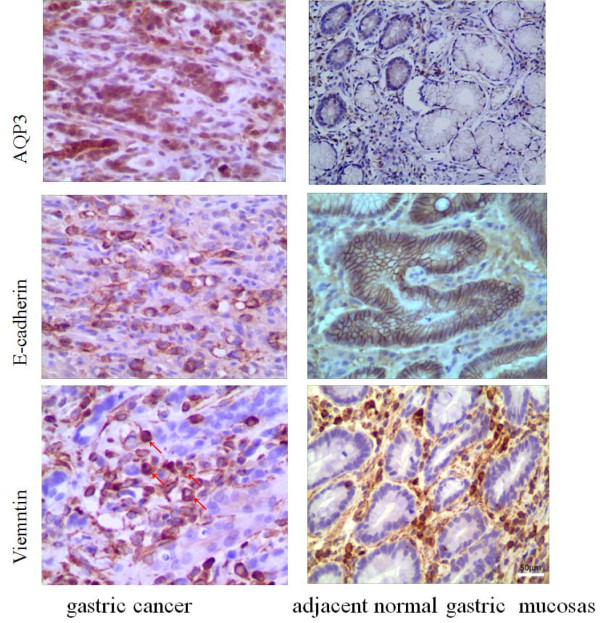
**Detection of AQP3, E-cadherin, and vimentin expression in GC tissue and adjacent normal tissue by IHC.** Strong AQP3 immunoreactivity was identified in poorly differentiated adenocarcinomas. E-cadherin expression was observed in normal gastric glands but not in GC tissue. Vimentin expression was not seen in normal tissue but was observed in GC tissue (red arrows). Original magnification, ×400.

As shown previously, GC tissues expressed significantly higher levels of AQP3 relative to normal gastric mucosa (Table 2, Figure 1). Expression of E-cadherin was down-regulated in GC tissues with respect to normal mucosa (*P* < 0.05) (Table 2, Figure 1). Positive signals for nuclear vimentin were detected in 15.7% (14/89) of cases, with vimentin only expressed in carcinoma tissues that over-expressed AQP3 and lacked expression of E-cadherin. Vimentin expression was not detected in normal gastric glands (Figure 1). The correlation between clinicopathological features in GC patients and expression of E-cadherin and vimentin was evaluated (Table 1). Elevated AQP3 expression in cancer tissues was associated with Lauren classification, lymph node metastasis, and lymphovascular invasion (*P* < 0.05). Lower levels of E-cadherin expression were closely related to depth of tumor invasion, lymph node metastasis, and lymphovascular invasion (*P* < 0.05). Vimentin expression was significantly associated with Lauren classification, depth of tumor invasion, and lymphovascular invasion (*P* < 0.05).

**Table 2:** Expression of AQP3 and E-cadherin in GC tissues and corresponding normal gastric mucosa tissues

**Proteins**	**Gastric cancer tissues**	**Gastric normal mucosa tissues**	***X***^**2**^	**P-value**
AQP3				0.000
Positive	65	27	32.486	
Negative	24	62		
E-cadherin				0.000
Positive	35	62	16.515	
Negative	54	27		

**Figure 2:**
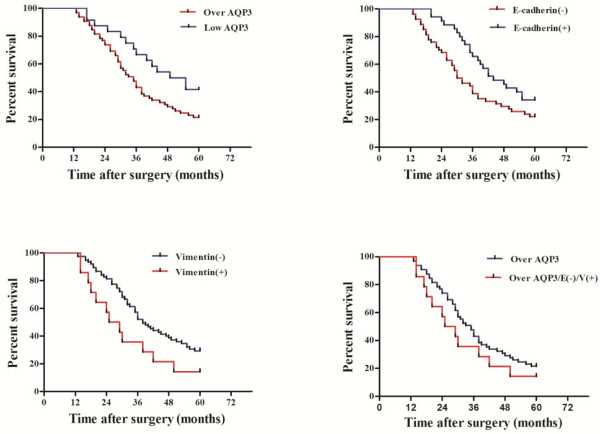
**Expression of AQP3 and associated EMT proteins predict poor prognosis of GC.** Patients that overexpressed AQP3 demonstrated shorter OS than those in the low expression group (*P* = 0.038). Patients with lower expression levels of E-cadherin had a worse OS than those with high E-cadherin expression levels (*P* = 0.008). Patients that were positive for vimentin expression exhibited poor survival rates compared with those who were negative for vimentin (*P* = 0.048). Patients with high expression levels of AQP3 but lacked E-cadherin and vimentin had a worse OS (*P* = 0.028).

The OS of the 89 patients in our study was 36 months. Based on IHC results, patients could be divided into different subgroups. Patients with AQP3 over-expression exhibited shorter OS than those in the low expression group (median OS time, 35 and 52 months, respectively; P =0.038; Figure 2). Patients with lower expression levels of E-cadherin had a worse OS than those with positive expression (median OS time, 31 and 44 months, respectively; P =0.008). Patients that were positive for vimentin expression exhibited a poor survival rate compared with the negative group (median OS time, 27 and 38 months, respectively; P =0.048). Among patients with high expression levels of AQP3, the subgroups that lacked E-cadherin and vimentin expression had a worse OS (median OS time, 27 and 35 months, respectively; P = 0.028). AQP3 over-expression, E-cadherin repression, and vimentin expression in GC could serve as factors predicting poor survival. AQP3 expression positively correlated with vimentin expression in GC tissues, but was inversely correlated with E-cadherin expression (*P* < 0.05; Table 3). Taken together, these findings indicate that AQP3 might be involved in the induction of EMT in GC.

**Table 3:** Correlation between expression levels of AQP3, E-cadherin, and vimentin in GC tissues by IHC

	**AQP3**			
	**+**	**-**	**r**	**P-value**
E-cadherin				
+	21	14	-0.236	0.031
-	44	10		
Vimentin				0.018
+	14	0	0.193	
-	51	24		

### AQP3 modulates cell proliferation, migration, and invasion of GC cells in vitro

**Figure 3:**
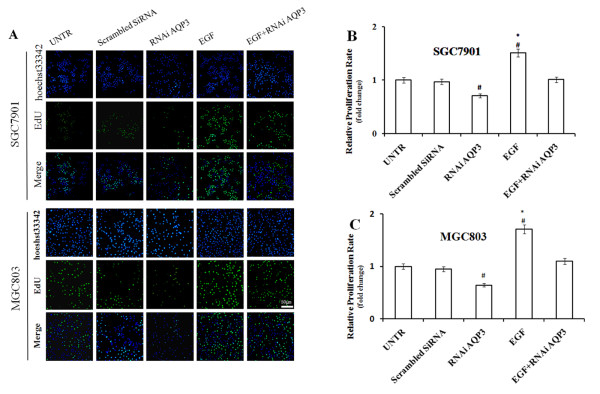
**AQP3 promotes cell proliferation of GC cells.** Cell proliferation of SGC7901 **(A and B)** and MGC803 **(A and C)** was significantly increased after treatment with EGF and decreased after treatment with RNAi AQP3. Data are expressed as the mean ± SE from three independent experiments. #*P* < 0.05 compared with the untreated group (UNTR); *P < 0.05 compared with the RNAi AQP3 group.

**Figure 4:**
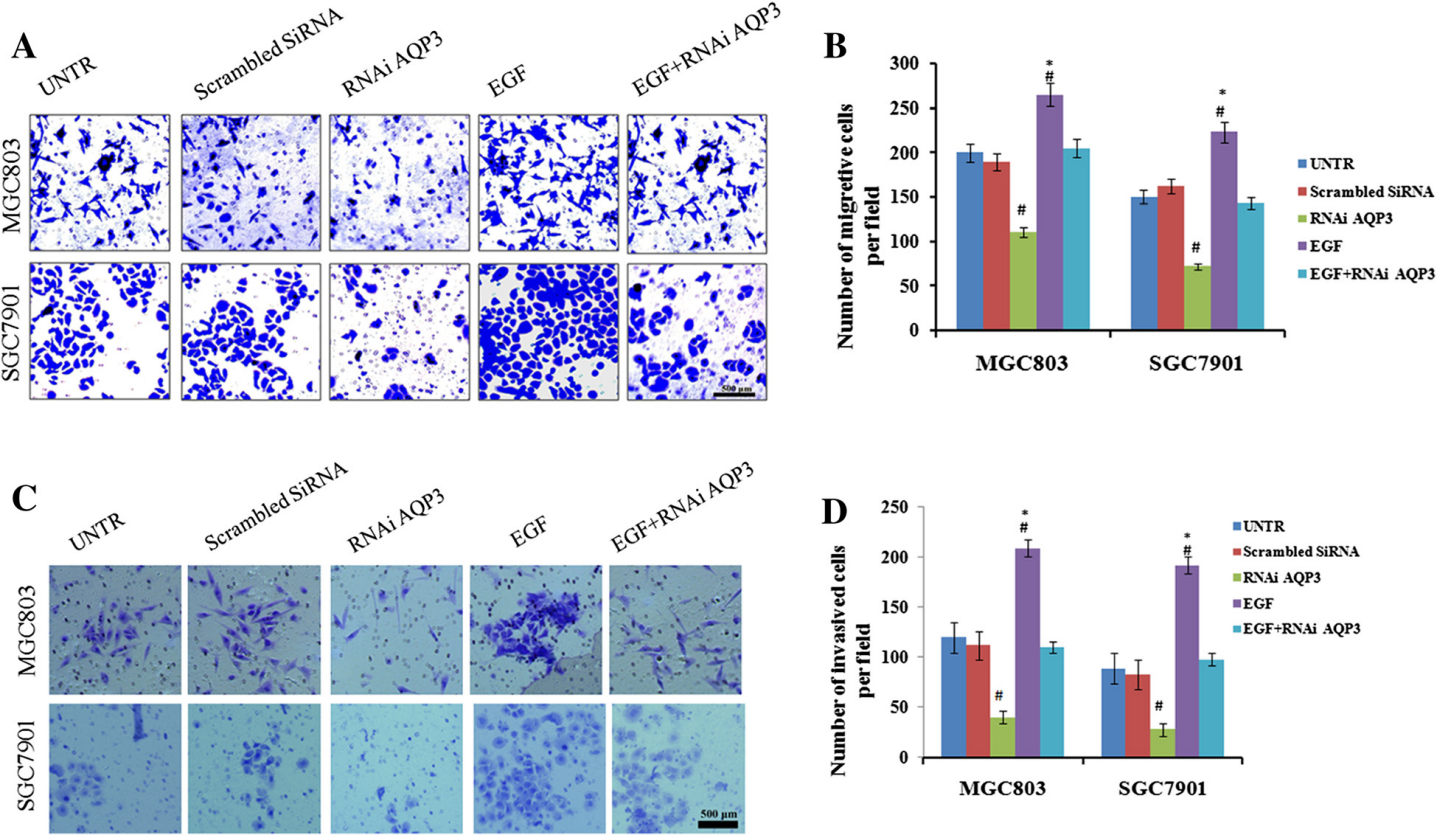
**AQP3 facilitates GC cell migration and invasion.** GC cell migration and invasion were detected using transwell migration and invasion assays. The number of cancer cells migrating through the Matrigel decreased significantly after treatment with RNAi AQP3 compared with the UNTR group, while treatment with EGF had the opposite effect **(A and B)**. AQP3-silenced GC cells invaded significantly slower when compared with the UNTR group and over-expression of AQP3 accelerated cell invasion **(C and D)**. Data are expressed as the mean ± SE from three independent experiments. #*P* < 0.05 compared with the untreated group (UNTR); *P < 0.05 compared with the RNAi AQP3 group. Original magnification × 100.

The proliferation of SGC7901 and MGC803 cells was significantly increased upon AQP3 over-expression, and significantly decreased after silencing of endogenous AQP3 (Figure 3), indicating that AQP3 enhances the proliferation of GC cells. When endogenous AQP3 was inhibited, the number of cancer cells migrating through matrigel was significantly decreased compared with the untreated group, while AQP3 over-expression had the opposite effect (*P* < 0.05; Figure 4). We also observed that AQP3-silenced GC cells invaded at a slower rate compared with the UNTR group (*P* < 0.05). Under the same conditions, over-expression of AQP3 accelerated cell invasion (*P* < 0.05). Our findings imply that AQP3 facilitates GC progression.

### AQP3 induces EMT of GC cells in vitro

**Figure 5:**
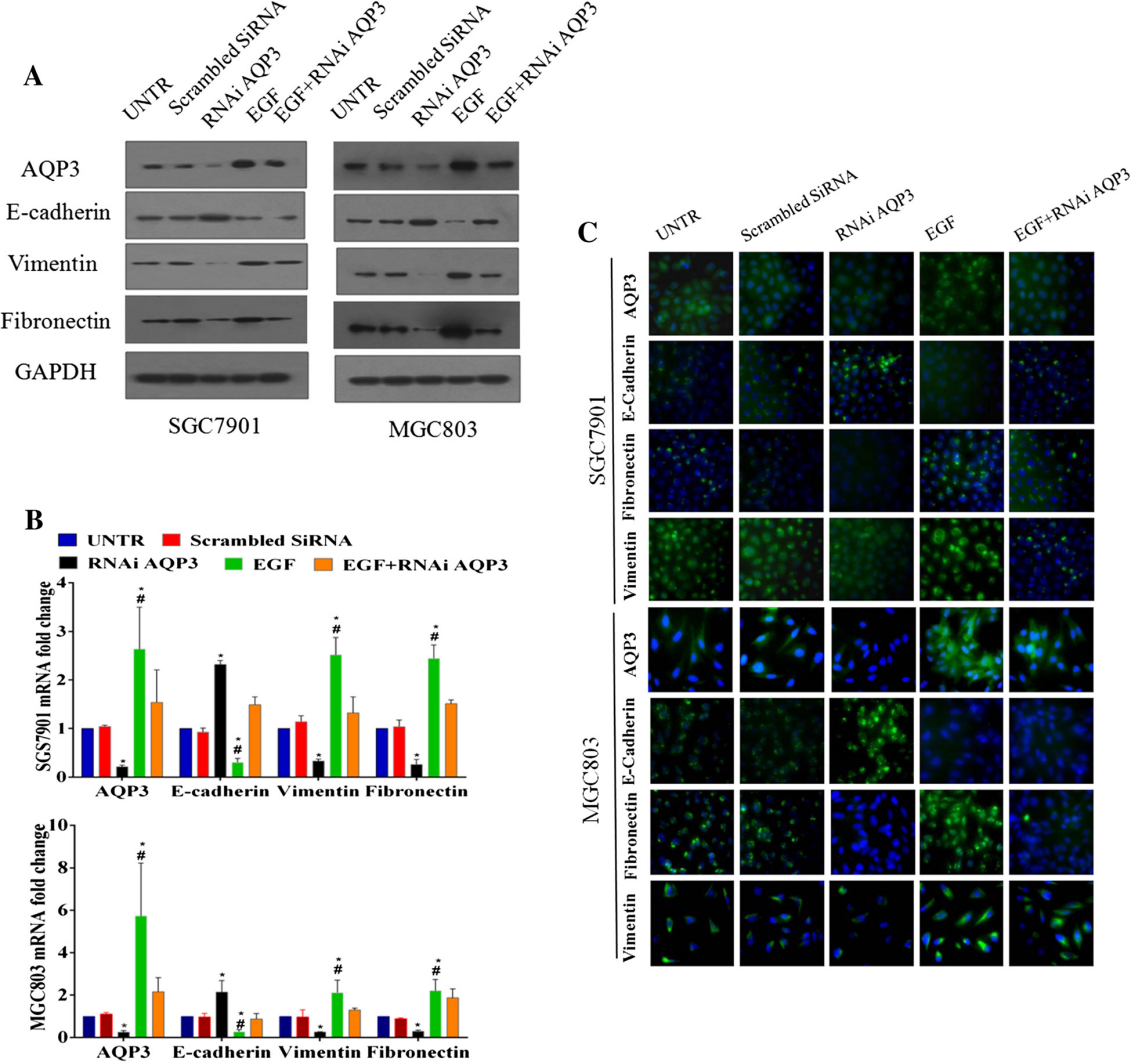
**AQP3 promotes EMT induction in human gastric adenocarcinoma cells. (A)** Expression levels of AQP3, E-cadherin, vimentin and fibronectin in SGC7901 and MGC803 cells were determined using western blots. GAPDH was used as an internal control. The relative accumulation of proteins in different groups was compared with those in the untreated group (UNTR). **(B)** mRNA expression levels of AQP3 and EMT-related proteins were assayed using qPCR. Data are expressed as the mean ± SE from three independent experiments. *P < 0.05 compared with the UNTR group; ^#^*P* < 0.05 compared with the RNAi AQP3 group. **(C)** Immunofluorescence assays for the detection of AQP3 and three EMT-related proteins. Target proteins were detected using the appropriate antibodies (green), and nuclei were stained with Hoechst33342 (blue).

We used siRNAs against AQP3 (RNAi AQP3) and EGF to down-regulate or up-regulate the expression of AQP3 in SGC7901 and MGC803 human GC cells. Expression of AQP3, E-cadherin, vimentin, and fibronectin was quantified by western blotting and qPCR. Compared with the untreated group, mRNA and protein levels of vimentin and fibronectin in cells over-expressing AQP3 were significantly increased, but decreased in AQP3-silenced cells. Expression levels of E-cadherin in cells overexpressing AQP3 were markedly decreased, but increased in AQP3-silenced cells (Figure 5A and B). The effect of AQP3 on expression levels of EMT-related proteins was confirmed by immunofluorescence staining (Figure 5C). These in vitro results suggest that the progression-promoting effect of AQP3 could be attributed to EMT induction of human GC cells.

### AQP3 regulates EMT in GC via the PI3K/AKT/SNAIL signaling pathway

**Figure 6:**
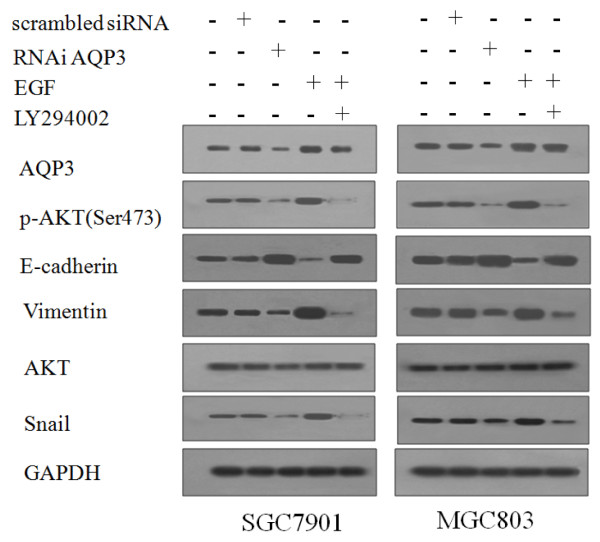
**AQP3 regulates EMT via the PI3K/AKT/Snail pathway.** SGC7901and MGC803 cells were treated with control siRNA, RNAi AQP3 and EGF, with or without a PI3K/AKT inhibitor. Proteins were analyzed by western blotting assay. GAPDH was used as an internal control. The relative accumulation of proteins was compared with the untreated group.

To test whether the PI3K/AKT pathway was involved in AQP3-mediated EMT, we examined the effects of AQP3 on PI3K/AKT activation and Snail expression. Phosphorylated AKT (p-AKT) was enhanced by AQP3 over-expression with EGF treatment, and was impaired by AQP3 down-regulation with RNAi AQP3 in SGC7901 and MGC803 cells (Figure 6). Expression of Snail was significantly increased with AQP3 over-expression, and decreased with AQP3 down-regulation. Phosphorylation of AKT was significantly inhibited by LY294002 in cells treated with EGF. Inhibition of p-AKT by LY294002 attenuated AQP3-induced Snail expression in cells. This initial study provides evidence that the PI3K/AKT/Snail signaling pathway is likely involved in AQP3-mediated EMT of human GC cells.

## Discussion

AQP3 has been established as a critical determinant of tumor growth and spread of human GC in previous studies. It has been speculated to promote GC cell migration and metastasis by inducing EMT. We found that AQP3 was up-regulated, and E-cadherin was repressed in cancer tissues. Vimentin immunoactivity was observed in 14 carcinoma tissues where AQP3 was overexpressed and E-cadherin was lacking. Over-expression of AQP3 correlated with repression of E-cadherin, and expression of vimentin. Loss of E-cadherin is regarded as a key step of EMT [24], while vimentin is a marker of mesenchymal differentiation [25]. EMT is thought to be transient and occurs during progression towards metastases in several types of solid tumors [22]. Our findings suggest that AQP3 is associated with EMT induction in human GC cases.

With respect to the clinical significance of AQP3 over-expression, E-cadherin repression, and vimentin expression, we showed that they were all associated with lymphovascular invasion. In particular, AQP3 and E-cadherin were associated with lymph node metastasis, while AQP3 and vimentin were associated with Lauren classification, and E-cadherin was associated with depth of tumor invasion. Patients with AQP3 over-expression exhibited worse OS compared with those lacking AQP3 expression. Repression of E-cadherin, and vimentin expression predicted poor prognosis for GC. These results are consistent with those reported by Zhou [25] and Corso [26]. However, our findings demonstrate for the first time the role of AQP3 in the prognosis of patients with GC.

Our previous results have shown that AQP3 promotes GC cell proliferation and migration. Because EMT of tumor cells is accepted to be closely associated with cancer invasion and metastasis [10,11], we investigated the effects of AQP3 on GC cell proliferation, migration, and invasion using EdU incorporation assays and transwell assays. AQP3 over-expression enhanced cell proliferation, migration and invasion, implying that AQP3 has a role in facilitating GC progression.

To determine whether AQP3 promotes GC progression through the induction of EMT, we investigated the effects of AQP3 on the expression of E-cadherin, vimentin, and fibronectin (all EMT-related proteins) in human GC cells. Expression of E-cadherin was down-regulated upon AQP3 over-expression, and up-regulated upon AQP3 silencing. Additionally, expression levels of mesenchymal markers (vimentin and fibronectin) correlated with AQP3 expression, suggesting that AQP3 is capable of inducing EMT in human GC. We postulated that the effects of AQP3 could be attributed to its induction of EMT in cases of human GC.

PI3K signaling plays a key role in inducing and maintaining EMT. Cells expressing a constitutively active form of PKB/AKT, the most important downstream effector of PI3K signaling, induces the expression of Snail-1, which in turn represses E-cadherin gene transcription and induces EMT [10]. In the present study, we showed that AQP3 over-expression enhanced the phosphorylation of AKT in cells, whereas AQP3 down-regulation had the opposite effect. Consistently, the expression of Snail correlated with AQP3 expression levels. A specific PI3K/AKT inhibitor attenuated AQP3-induced phosphorylation of AKT and Snail expression. These preliminary results reveal that the PI3K/AKT/Snail signaling pathway is likely involved in AQP3-mediated EMT of human GC cells.

## Conclusions

In conclusion, the collective findings from our study suggest AQP3 predicts poor prognosis in patients with GC, and that AQP3 promotes EMT in human GC cases via the PI3K/AKT/Snail signaling pathway. Our observations have further characterized the role of AQP3 in human GC, increasing the likelihood that AQP3 could be exploited as a potential diagnostic and prognostic biomarker of GC progression, and provide an important target for therapeutic intervention.

## Competing interests

The authors declare they have no conflicts of interest.

## Authors’ contributions

LZS conceived and designed the experiments. JC, TW and YCZ performed the experiments. JC, TW, YCZ and FG analyzed the data. ZHZ, HX and SLW supervised the whole experimental work and revised the manuscript. JC, TW, YCZ and LZS wrote the paper. All authors read and approved the manuscript.
